# Addressing the role of medical students using community mobilization and social media in the Ebola response

**DOI:** 10.1007/s40037-016-0271-7

**Published:** 2016-05-23

**Authors:** Helena J. Chapman, Victor J. Animasahun, Adesoji E. Tade, Asad Naveed

**Affiliations:** Department of Environmental and Global Health, University of Florida, Gainesville, Florida USA; International Federation of Medical Students’ Associations, Amsterdam, The Netherlands; Faculty of Clinical Sciences, Obafemi Awolowo College of Health Sciences, Olabisi Onabanjo University, Sagamu, Nigeria; Faculty of Clinical Sciences, College of Medicine and Allied Health Sciences, University of Sierra Leone, Freetown, Sierra Leone

**Keywords:** Ebola virus disease, Social media, Health promotion, Medical education

## Abstract

Health professions education in the 21st century should incorporate both community mobilization and social media strategies. First, community mobilization facilitates change by educating community members with evidence-based, high-quality and up-to-date health information and empowering their active participation in target health initiatives. Second, advancements in technology and globalization foster the development of innovative communication technologies used as a key tool in the ‘roll out’ of community health initiatives during epidemics such as Ebola virus disease. In August 2014, medical students of Sierra Leone and Guinea used these dual health promotional strategies in the Kick Ebola Out campaign to educate community members about transmission of the Ebola virus and preventive measures, as well as to reduce perceptions related to stigma or fear of disease transmission. In this report, we describe how medical students, who are trained in basic and clinical sciences, evidence-based practices, and social determinants of health, can serve as human resources for health and facilitate dynamic communication strategies to educate and empower both medical students and community members for local or national health initiatives.

Over the past decade, Web 2.0 applications, defined as internet-based tools that allow users to generate and manage content, have demonstrated the potential to revolutionize the search of up-to-date health information by users and the respective dissemination by health professionals [1]. Considered the ‘social Web’, this updated web-based platform facilitates personable interactions between individuals, whether sharing educational or entertainment information (e. g. YouTube, Instagram, blogs, podcasts) or networking (e. g. Facebook, Twitter, Google Plus) [2]. Traditional health promotion tools, such as television or radio broadcasts, newspapers or pamphlet distribution, used in mass media campaigns have demonstrated positive influences on health behaviours [3]. By incorporating these technological advances of the ‘social Web’ into traditional health promotion tools, health teams can develop local or national primary, secondary or tertiary prevention campaigns that improve dissemination of health information to available users. This potential benefit of the use of social media technology in campaigns has been demonstrated in a review of case studies [4].

‘Social mobilizers’, on the other hand, are key to implementing local or national health initiatives in the field and building rapport with community members [5]. Although no standard definition of community mobilization exists [6], this notion refers to facilitating change through educating community members to increase their knowledge and skill sets, empowering active participation in programmed activities, and forming partner collaborations at local or national levels [7, 8]. When highly trained in project objectives and activities, these community health workers, who understand the local cultural beliefs or customs, can reach marginalized communities and teach accurate health information [6]. When joined with interdisciplinary health teams, they can tailor the health message to be culturally appropriate, increase interpersonal communication that empowers community members, and promote optimal health outcomes [9]. It is anticipated that community members will use recommended primary prevention methods, such as understanding risk factors or immunizations, which mitigate disease transmission. This was observed during the Ebola epidemic in Western Africa in 2014, where ‘social mobilizers’ visited homes, interacting with individuals and families to raise community awareness and reduce stigma associated with the transmission of the Ebola virus [10].

Although ‘social mobilizers’ play a significant role in the field, there is limited research that describes the role of medical students when using the dual strategies of community mobilization and social media. Medical students, from their health professions education, understand the influence of the social determinants of health as well as the basic anatomy, physiology and epidemiology of disease pathology and transmission. They are creative and highly skilled in the use of social media technology, such as Facebook, Google Plus, Instagram, Twitter, or YouTube, to develop catchy slogans, hashtags, or taglines that are popular and tailored to affected communities. Thus, they may be trained and serve as essential team members in the ‘roll out’ of health educational outreach programmes during disaster relief efforts.

We describe how African medical students in Sierra Leone and Guinea applied dual community mobilization and social media approaches during the Ebola epidemic. We also provide reasons that support the inclusion of medical students, due to their training in basic and clinical sciences and skills in Web 2.0 applications, on intra- or multi-disciplinary health teams during relief programmes.

## The case of the Ebola virus

The emergence of Ebola virus disease in Guinea in March 2014, followed by Liberia and Sierra Leone [11], provided optimal conditions for disease transmission. This resulted from the three neighbouring countries having fluid borders; high human travel between rural, peri-urban, and urban regions or countries; and collapse of environmental and health systems during post-civil war rebuilding [12]. Initially, the rapid communicability and high mortality rates of Ebola virus disease presented multiple challenges to clinicians, health facilities, and national health sectors. Suboptimal isolation facilities for Ebola virus disease were widespread. Health facilities were understaffed, with less than an estimated 0.1 physicians per 1,000 population in these three respective countries [13]. These burdened health care workers encountered complex challenges in managing the increased number of suspected patients with Ebola virus disease while diagnosing those with other infectious diseases of similar clinical presentation, such as cholera, dengue, malaria, or typhoid fever.

International public health authorities, such as the World Health Organization (WHO), distributed posters and pamphlets [14], and local Ministries of Health and Sanitation conducted radio and television broadcasts to increase community awareness about Ebola virus disease [15, 16]. Widespread public media messages emphasized adherence to standard protocols, basic hygiene policies, and quarantine measures or *cordons sanitaires*, in addition to eliminating specific cultural practices that facilitated physical contact with infected individuals. Understanding the cultural role of oral communication in Liberia, for example, ‘social mobilizers’ entered local communities to channel accurate health information about Ebola virus disease [17]. It is essential that health interventions, such as infection control practices, are precisely aligned with cultural practices to maximize community acceptance [18]. In the presence of this ‘intervention–culture’ disconnect, some authorities observed cultural backlash from community members with escalating levels of distrust and fear of relief responses to Ebola virus disease, including stigma associated with strict isolation procedures. Thus, lessons learned were the key intersections among the identification of cultural beliefs, appraisal of existing health systems, use of evidence-based practices by interdisciplinary teams, and household- and community-level participation [19, 20]. Understanding the intertwining roles of these constructs may serve as a foundation to establish and strengthen cultural-based, standardized protocols for infection control during relief programmes, such as for Ebola virus disease.

## Utilized health educational strategies in a case study

**Fig. 1:**
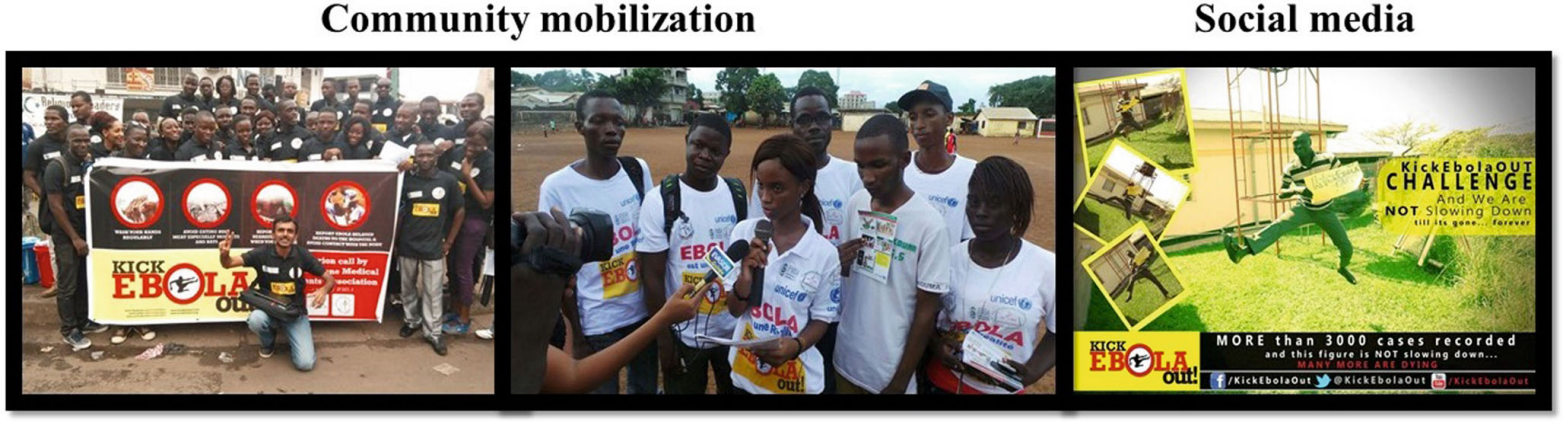
Dual health promotional strategies of community mobilization and social media used by medical students during the Ebola virus disease outbreak in Sierra Leone and Guinea in 2014

Due to the temporary closure of educational institutions in Sierra Leone and Guinea, including one medical school in Sierra Leone and three medical schools in Guinea, medical students of all academic years, forming part of the International Federation of Medical Students’ Associations (IFMSA), initiated the Kick Ebola Out campaign in August 2014 [21]. Since medical students learn the basic and clinical sciences with community and clinical rotations with patients in health institutions, their knowledge and skill sets are more advanced than other community health workers. Through didactic sessions, they were trained by specialists of the respective Ministries of Health and Sanitation on updated knowledge about Ebola virus disease and how to dispel common myths or misinformation. Representing multiple ethnicities, they were also instructed on appropriate and culturally sensitive communication strategies to use in community campaigns, including disseminating health messages through social media. By using dual health promotional strategies of community mobilization and social media, medical students could address two main objectives of the campaign (Fig. 1). The first objective was to increase overall knowledge of medical students and community members about Ebola virus disease transmission, clinical presentation and preventive measures. The second objective was to reduce community perceptions related to Ebola-associated stigma or fear of disease transmission for susceptible individuals or recent survivors.

Using community mobilization strategies, groups of medical students, who were led by at least one advanced medical student, conducted home visits to numerous communities, distributed chlorine and soap, and provided accurate health information about Ebola virus disease. By visiting media outlets, such as television and radio stations, they educated audiences about Ebola virus disease and dispelled myths without provoking alarm or stigma to transmission of the Ebola virus. They discouraged handshaking as well as certain cultural practices, such as washing or touching the bodies of patients infected with Ebola virus disease. Still, failure to abide to the established quarantine protocol due to fear or anxiety, lack of trust in orthodox medical practice, low literacy levels, and extreme poverty in communities challenged Ebola containment measures [22].

The advantage of integrating social media, such as Facebook and Twitter, in the Ebola virus educational campaigns was twofold. First, these Web 2.0 applications facilitated rapid communication among medical students of multiple African countries and other geographic regions. Professional networks were solidified, accurate Ebola virus disease information was identified, and health promotional tools (@KickEbolaOut) and an educational mobile telephone app (KickEbolaOut) were created and shared. Second, by using this virtual interface to disseminate key health facts and graphics on Ebola virus disease, community members could read up-to-date health messages and, in turn, educate family members. For example, Google and Twitter movements increased when newsworthy findings were promoted on media outlets, such as when new cases of Ebola virus disease were identified in the United States [23].

One limitation of this campaign was the possible distrust or fear of community members, related to the stigma associated with Ebola virus disease, which influenced their ability to participate in educational activities during home visits. Medical students reported that due to their identity as health professional students, they were positively received and welcomed by community members. A second limitation was misinformation that spread quickly through social media, because it was not possible to validate or verify the quality of millions of Ebola tweets or posts [24]. To combat this challenge, African medical students spearheaded the development of Twitter handles, Facebook pages and posts, and hashtags, which enabled prompt dissemination of accurate messages from reputable health authorities, including WHO (@WHO), Centers for Disease Control and Prevention (@CDCgov), and Médecins Sans Frontières (@MSF). Because social media technology spans across all age groups and socioeconomic strata, this veritable tool in health promotion can significantly aid health teams in disseminating accurate information among health care providers and community members during outbreak relief efforts.

## Conclusions

As described in this report, medical students can serve as human resources for health through their integral role in disseminating health information and achieving health programme objectives through the use of community mobilization and social media strategies. Due to their knowledge and skills in medicine, public health and Web 2.0 applications, they can critically evaluate evidence-based health messages for accuracy, utilize cultural-sensitive communication strategies to relay health messages and develop creative messages that can be publicized on radio and television broadcasts and social media for communities. The power and structure of the IFMSA national member organizations worldwide provide an effective pathway and approach to facilitate Ministry of Health and Sanitation training of medical students on key clinical and communication skills. By using these dual health promotion strategies, medical students can join intra- or multi-disciplinary health teams and establish innovative health programmes for primary, secondary or tertiary health initiatives at local or national levels.

The role of medical students should be recognized as an indispensable health educator in community-based health promotion and education programmes. Due to their vibrancy, passion, and expertise in the use of social media strategies, they serve as key team members to facilitate broad diffusion of health messages that keep community members educated and empowered for their optimal health decisions. Although academically prepared with their health professions education in the classroom, they may have limited opportunities to apply this acquired knowledge and skill set in the community setting. Therefore, the future integration of early practical community experiences utilizing social media strategies throughout the health professions education may strengthen academic training of medical students in public health, as well as provide them with a specialized toolkit to use when leading local or national health initiatives, such as the relief programmes during the Ebola outbreak.
